# Forecasting photovoltaic power in high-latitude regions via support vector machine optimized by meta-heuristics

**DOI:** 10.1038/s41598-025-33415-7

**Published:** 2026-01-06

**Authors:** Sertaç Oruç, Mehmet Ali Hınıs, Türker Tuğrul

**Affiliations:** 1 The Center for Sámi Studies, UiT Norges Arktiske Universitet, Tromsø, 9037 Norway; 2 Civil Engineering Department, Faculty of Engineering and Natural Sciences, Ankara Yıldırım Beyazıt University, 15 Temmuz Şehitleri Campus, Ankara, 06010 Turkey; 3 Civil Engineering Department, Faculty of Engineering, Central Campus, Aksaray University, Aksaray, 68100 Türkiye Turkey; 4 Civil Engineering Department, Technology Faculty, Central Campus, Gazi University, Ankara, 06560 Turkey

**Keywords:** Photovoltaic power forecasting, Machine learning, Metaheuristic optimization, Solar energy, Energy science and technology, Engineering, Mathematics and computing

## Abstract

Machine-learning techniques are widely used across many disciplines, including electricity generation forecasting. In this study, the Support Vector Machine (SVM) based models, one of the machine learning techniques, were developed for daily PV power forecasting. To improve model performance, models were tuned with four metaheuristic optimizers, including the Artificial Bee Colony (ABC), Grey Wolf Optimizer (GWO), Genetic Algorithm (GA), and Particle Swarm Optimization (PSO). Daily PV power and temperature data from 2020 to 2023 were obtained for the Stavanger, Oslo, and Kristiansand regions which located in southern Norway. One of the innovative aspects of this study is the investigation of the performance of SVM (Support Vector Machine) combined with various optimization methods across four alternative input configurations. To examine the different feature combinations, four different input configurations were created through the Minimum-Redundancy Maximum-Relevancy (MRMR) method. The analysis results obtained with SVM were further enhanced using all optimization techniques. Among those, the SVM-PSO-M04 (*r* = 0.7707, NSE = 0.5748, KGE = 0.7092, PI = 0.2964 and RMSE = 0.6513) method produced the most effective results (improving the correlation coefficient (r) to 0.7707 (approximately a 19% increase over the untuned SVM)) among the tested hybrid configurations obtained in our experiments. Moreover, coupling temperature data alongside PV power as model input also tends to improve forecasting skill. Results of this study provide a case-study benchmark for researchers, institutions, and other stakeholders engaged in renewable energy planning and management in high-latitude regions.

## Introduction

Solar energy has emerged as a critical frontier of global decarbonisation strategies. Yet, under climate change, the power capacity of these systems remains fundamentally linked to meteorological conditions that are themselves undergoing a transformation^1–5^. Long term climate model ensembles consistently project regional scale shifts in both surface solar radiation and near-surface air temperature which jointly determine photovoltaic (PV) efficiency^6–10^. While solar energy is subject to geographic and temporal variations^4,6^, the relation between rising temperatures and solar energy production introduces a more complex technical challenge. The linear degradation of photovoltaic conversion efficiency as cell temperature rises is a fundamental issue particularly in urban or low-latitude regions which consequently can offset the benefits from increased insolation even with minor warming conditions^9,11,12^. A case study in Istanbul exemplifies this negative synergy. The study demonstrated that according to RegCM based projections through 2050, a small decrease in incoming radiation levels may pose a direct threat to future PV yields when coupled with simultaneous multi-degree warming^13^. For ensuring modern energy grids remain resilient in the face of a rapidly shifting climate accurate solar power forecasting is a fundamental necessity. 

Future climate conditions, intensified by climate change, are expected to increase the complexity of PV system from initial design through to operation, and maintenance. In Europe and parts of East Asia as the reduction in cloud cover is expected to offsets thermal performance losses, photovoltaic yields are expected to experience modest rise, even under aggressive warming scenarios^4,5,7,9,10^. Conversely regions like West Africa, North Africa, Australasia and parts of Central Asia are likely to face declines linked to solar dimming and rising cell temperatures, although most studies still find decreases remain within a 6% margin of current baselines^4,6,8,10,12^. While global case for solar remains as a no regrets investment, site specific planning must integrate a consistent climate signal into long-term energy planning to ensure sustained accuracy when projections are considered^1–3,5,7^.

Predictive analytics have become from a support tool to a fundamental need to maintain grid stability and manage net loads effectively as solar integration grows^14,15.^ Beyond immediate operations, solar forecasting plays an important role in the economic profile of projects by optimizing battery storage and reducing wasted energy through curtailments^16^. It is suggested that integrating solar predictions with storage systems not only lowers operational expenses but also decreases overall grid dependency^17^. While various modeling techniques, apart from traditional regression, such as advanced deep learning, offer reliable short- and long-term outputs^18,^ recent evidence suggests that the latter has demonstrated superior accuracy for irradiance forecasting^19.^Considering future climate where solar volatility is expected to intensify, the ability to quantify prediction uncertainty will be beneficial for microgrid resilience and the sophisticated management of modern power infrastructures^20^. Solar power prediction provides consistent grid operation, storage optimization, economic allocation, and integration of renewable sources. Furthermore, it helps in reducing the impacts of climate-induced variability. Ultimately, these forecasting tools serve as the backbone for consistent, climate-resilient power systems.

Recent research on solar-irradiance forecasting spans a wide spectrum of time-horizons and data sources, progressing from purely statistical baselines to highly integrated, image-enhanced deep-learning pipelines. Early work demonstrated the value of classic statistical approaches: Paulescu and Paulescu^21^ showed that an empirical two-state clear-sky model outperformed random-walk, moving-average and Autoregressive integrated moving average (ARIMA) baselines for four-samples-per-minute data from Timișoara, Romania, while Zambrano and Giraldo^22^ built multidimensional transfer models that dispense with costly on-site training measurements. Parallel semi-empirical efforts for hourly horizons combined extraterrestrial irradiance and clearness-index signals to surpass the Angström-Prescott formula at several Turkish sites^23^. Surveys and reviews have mapped the methodological landscape, covering statistical, cloud-image and NWP (Numerical Weather Prediction), routes^24^ and benchmarking time-series, image and hybrid families^25^.

Machine-learning studies have gradually pushed forecast granularity to the minute scale. Image-only pipelines link real-time sky-camera RGB profiles to 1–10 min global horizontal irradiance with competitive the mean absolute percentage error (MAPE) and the root mean square error (RMSE) scores^26^; all-sky imagers coupled with simultaneous irradiance readings achieved ramp-event detection indices of 43–62% at a Uruguayan test bed^27^. Satellite/NWP coupling also remains powerful at the 0–3 h range, trimming persistence errors by ≈ 10 W m⁻² across the U.S. SURFRAD network^28^. Where on-site imagery is unavailable, short-term physics-free predictors such as Artificial Neural Network (ANN)-SFP^29^ and daily-scale SVM/ANN/k-NN ensembles^30^ still yield R² values up to 0.94.

Deep learning has become the dominant trend for sub-hourly horizons. For example, CNN–LSTM hybrids (convolutional neural networks combined with long short-term memory networks) that fuse wavelet-packet-decomposed sequences with ground imagery have been shown to lower RMSE compared to back-propagation neural networks (BPNN), Support Vector Regression (SVR), and standalone LSTM models on three U.S. stations^31^, while multi-modal deep clustering aligns cloud-camera frames with the Numerical Weather Prediction (NWP) fields, reaching a 29.4 W m⁻² day-ahead RMSE in California^32^. Recurrent architectures remain strong: Deep Recurrent Neural Networks (DRNN) surpassed SVR and feed-forward networks in Canada^33^; LSTM networks delivered R² > 0.9 under complicated weather in Atlanta and Hawaii^34^; and bidirectional/attention LSTMs benefited from multi-site NASA POWER inputs across India^35^. Cutting-edge transformer variants now integrate variational-mode-decomposed components, eclipsing Convolutional Neural Network (CNN)-LSTM and vanilla transformer baselines over a 2015–2019 EMAP data set^36^. Comprehensive Indian reviews confirm that such CNN, LSTM and CNN-LSTM hybrids can lift accuracy by up to 37%^37^.

Collectively these studies highlight three converging insights for solar-radiation forecasting. First, hybridization, whether statistical-empirical, image-plus-NWP, or signal-decomposition-plus-deep-network, consistently boosts performance across climates. Second, model choice and horizon must reflect data availability: transfer learning and clear-sky filters remain valuable when imagery is scarce, whereas minute-ahead dispatch benefits most from sky cameras and CNN-LSTM fusion. Third, the field is shifting toward interpretable, multi-modal deep architectures that can generalize without local retraining, a direction reinforced by the superior accuracy of multi-modal deep clustering (MMDC)^32^ and Multiple Image Convolutional Long Short-Term Memory Fusion Network (MICNN-L)^25^. Remaining gaps include systematic cross-climate validation beyond the U.S., Europe and India, and unified uncertainty quantification to complement RMSE-based metrics.

In addition to new DL techniques and ML methods, nature-inspired metaheuristics have also emerged as powerful tools for solving complex nonlinear, multimodal optimization problems, particularly in the domains of machine learning model tuning, feature selection, and system design. Among these, the Artificial Bee Colony (ABC), Grey Wolf Optimizer (GWO), Genetic Algorithm (GA), and Whale Optimization Algorithm (WOA) are widely recognized for their balance of exploration and exploitation strategies. Those optimization algorithms are used both as a single optimization technique or a combination of various alternatives.

ABC algorithm, inspired by the foraging behavior of honeybees, has gained attention due to its simplicity and strong global search capabilities. As a population intelligent algorithm, ABC applied in many studies. Oruc et al.^38^, Zhang et al.^39^, Gujarathi et al.^40^ incorporated ABC directly employed with a ML method or hybridized with another optimization algorithm in studies ranging drought forecast to engine optimization and handling with high dimensional datasets. GWO mimics the social leadership and cooperative hunting strategies of grey wolves. It is valued for its algorithmic simplicity and convergence stability. GWO has been successfully applied in time series forecasting and parameter tuning tasks and demonstrated its effectiveness in optimizing^41–43^. GA is a classical evolutionary algorithm based on principles of natural selection, crossover, and mutation^44^. It has broad application across optimization tasks and is often used as a baseline for performance comparisons with newer methods. Genetic Algorithms remain to be used in hybrid models to boost convergence rates across problem spaces containing either discrete or continuous parameters in diverse application^44–47^. The Whale Optimization Algorithm (WOA) inspired from feeding strategy of whale population^48^. WOA gained recognition for its simplicity and ability to balance intensification and diversification. Tang et al.^49^ introduced a combined WOA-ABC algorithm to overcome local optima problem and improve solution accuracy across theoretical and practical engineering problems. Although less frequently cited in academic databases, the Coyote Optimization Algorithm (COA) or COATI represents another nature-inspired method that modeled on coyote pack dynamics and social learning^50^. Despite showing potential across broad domains ranging from energy planning to photovoltaic parameter extraction^51,52^, additional comparative studies are needed to validate its performance relative to better-established algorithms like ABC, GWO, and WOA. Similar to other algorithms, since its initial development, COA has evolved into several improved forms, including Chaotic COA (CCOA)^53^ and Multiobjective COA (MOCOA)^54^ which have been implemented in a broad range of problem domains.

As Norway targets toward broadening its energy mix and reduce its dependence on fossil fuels sources, solar power becomes the increasing component of the energy strategy. Norway now recognizes the importance of solar energy as an important complementary source of renewable electricity generation while it has been historically known for its hydropower capabilities^55–57^. The possibility of using solar energy across Norway’s diverse landscapes, such as urban areas, agricultural regions, and industrial sites, has become much more realistic due to the significant drop in solar PV system prices and progress in solar technology in recent years^56,58^. As solar energy will not be sufficient to supply Norway especially during the winter months, hybrid systems offer a promising solution for integrating solar energy into Norway’s energy landscape to meet the demands for renewable energy^59^. While Norway is not typically known for its solar energy production, it possesses a number of hidden advantages for PV performance. The favorable effect of low ambient temperatures on solar panel efficiency counterbalance lower irradiance levels by reducing heat-related efficiency losses since PV systems, especially those based on crystalline silicon, are thermally sensitive—their efficiency decreases by ~ 0.4–0.5% per °C increase in module temperature^59–63^. Moreover, new applications like icephobic nanocoatings^64^, considering climate variations and orientations affect^65^ increasing performance of the systems. Rees et al.,^66^ also built a simple, transparent workflow that turns freely available high-resolution LiDAR into city-wide rooftop-solar potential estimations and concluded that Rooftop solar PV in Tromsø could realistically supply ≈ 20–30% of the city’s annual electricity demand, about 200 GWh yr⁻¹, with residential roofs alone contributing roughly 40% of that total. In addition, new technologies, including tilting systems, bifacial panels, and heat recovery-integrated PV (PVT), present opportunities to improve year-round utilization^67–70^.

Given the evolving scientific landscape and Norway’s focus on solar energy, there is a need to integrate advanced solar power forecasting methodologies into regional energy planning and grid management. International studies have exhibited the superiority of hybrid deep learning models, especially those combining sky imagery, NWP outputs, and statistical decomposition. Though, local adoption in high-latitude contexts like Norway remains limited. The seasonal extremes in insolation, coupled with complex topography and urban form, demand forecasting frameworks that are both data-adaptive and climatically robust. Moreover, with evidence that rooftop solar in cities like Tromsø could meet up to 30% of local electricity demand, the importance of precise, site-specific irradiance forecasting becomes even more pronounced.

Many forecasting studies in environmental sciences and renewable-energy applications focus on a small set of established learning algorithms (e.g., ANN, SVM/SVR, and Random Forest) for which performance and generalization properties have been widely discussed^71^. In addition, several studies report that hybridization, through data preprocessing and/or metaheuristic hyperparameter tuning, can further improve SVM/SVR performance in related forecasting tasks^72–74^ Accordingly, the present study uses SVM as a controlled base learner to systematically quantify the incremental value of (i) feature selection and lag-structure design and (ii) different metaheuristic optimizers. The aim is not to claim universal superiority over all alternative machine-learning/deep-learning methods, but to provide a transparent, like-for-like comparison within a unified modeling framework.

In line with this objective, we develop and evaluate SVM-based hybrid forecasting models for daily PV power in southern Norway (Kristiansand, Stavanger, and Oslo) using PVGIS-ERA5 derived PV output and near-surface air temperature data covering 2020–2023. Four metaheuristic optimizers (ABC, GWO, GA, and PSO) are compared, and four alternative input configurations are defined via the Minimum Redundancy Maximum Relevance (MRMR) feature selection method. Model performance is assessed using multiple goodness-of-fit criteria (r, RMSE, NSE, KGE, and PI) together with visual diagnostics.

The main contributions of this work are as follows:

- A daily PV power forecasting case study for three southern Norway sites using consistent PVGIS-ERA5 input data.
- Development of a controlled comparison of four metaheuristic optimizers (ABC, GWO, GA, PSO) for tuning an SVM forecasting model.
- Construction of four lagged input structures via MRMR and evaluation of four lagged input structures combining PV history and temperature signals.
- Multi-metric evaluation (r, RMSE, NSE, KGE, PI) complemented by visual diagnostics to interpret performance differences and model limitations.

## Materials and methods

**Fig. 1:**
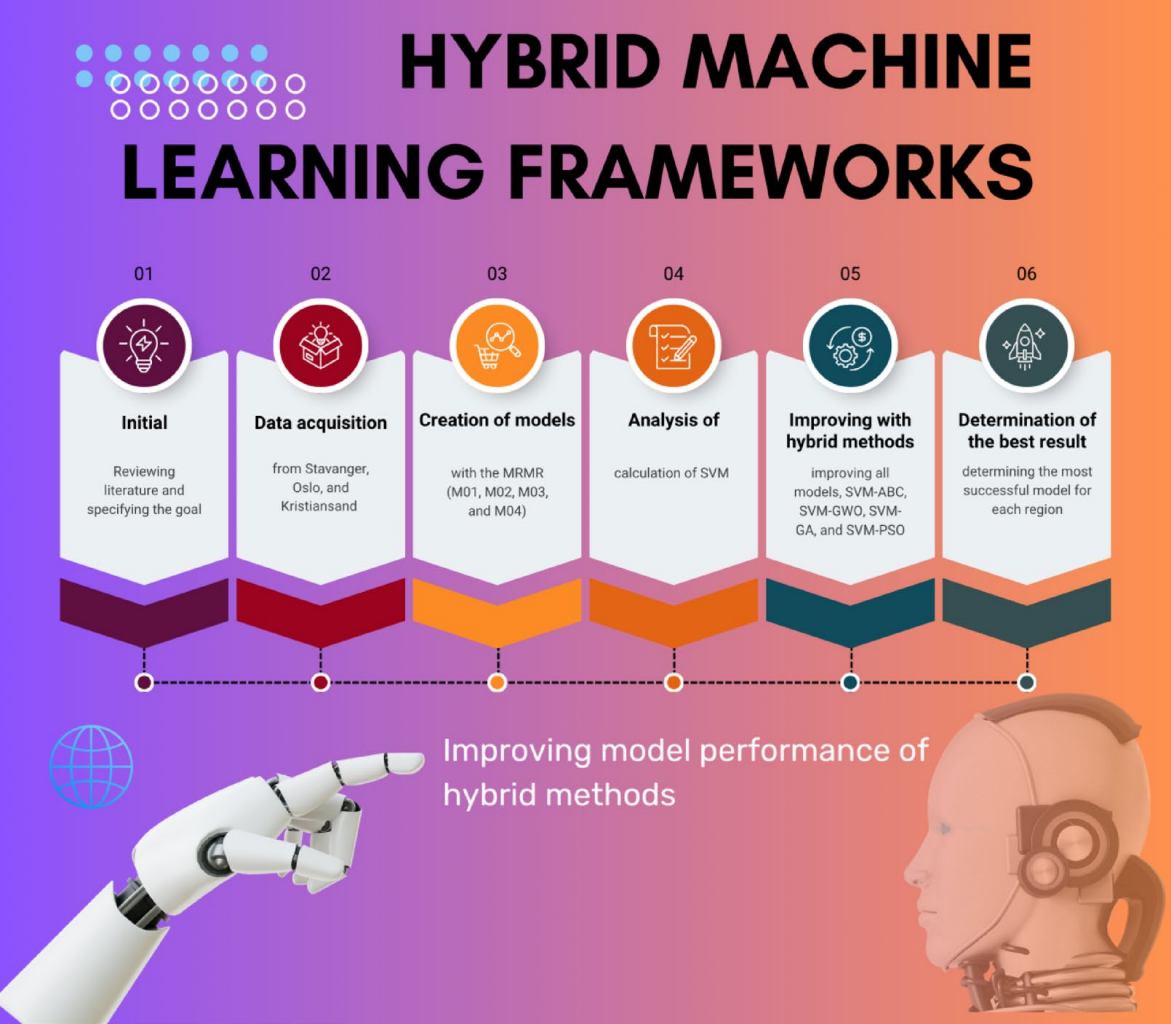
Methodological framework.

This work presents an analysis of PV generation prediction based on the data derived from three distinct locations of Norway. The analysis used SVM with a range of optimization techniques implemented including PSO, GWO, GA and ABC. The methodological framework in this study is summarized graphically in Fig. 1.

### Study area and dataset

This study focuses on three coastal locations across southern Norway to evaluate solar radiation and PV power potential. The selected sites vary in latitude, elevation, and orientation. This diversification provides examination of how geographic and topographic parameters affect solar energy production. Geographically, the study area extends between 58.15° to ~ 59.90°N latitude and 5.73° to ~ 10.74°E longitude which covers a diverse range of coastal terrains.

Table 1 presents the key site characteristics including geographic location, elevation above sea level, slope, azimuth, and PV system specifications considered. The PVGIS-ERA5 radiation database was used to ensure climatic input consistency across all locations (PVGIS 5.3)^75^. The optimal slope angles fall within 46° to 49 range and azimuth values span from − 5° to 0°. These values indicate minor deviations from due south alignment, which is considered the optimal for fixed PV installations at Northern Hemisphere latitudes.

A standard monocrystalline silicon PV system with a nominal capacity of 1.0 kWp were assumed for each site. Uniform system losses of 14% are applied to account for thermal, wiring, and inverter-related inefficiencies. The selection of these representative sites and configuration aim to make the results more comparable and transferable to broader PV deployment scenarios across similar latitudes and climates.

**Fig. 2:**
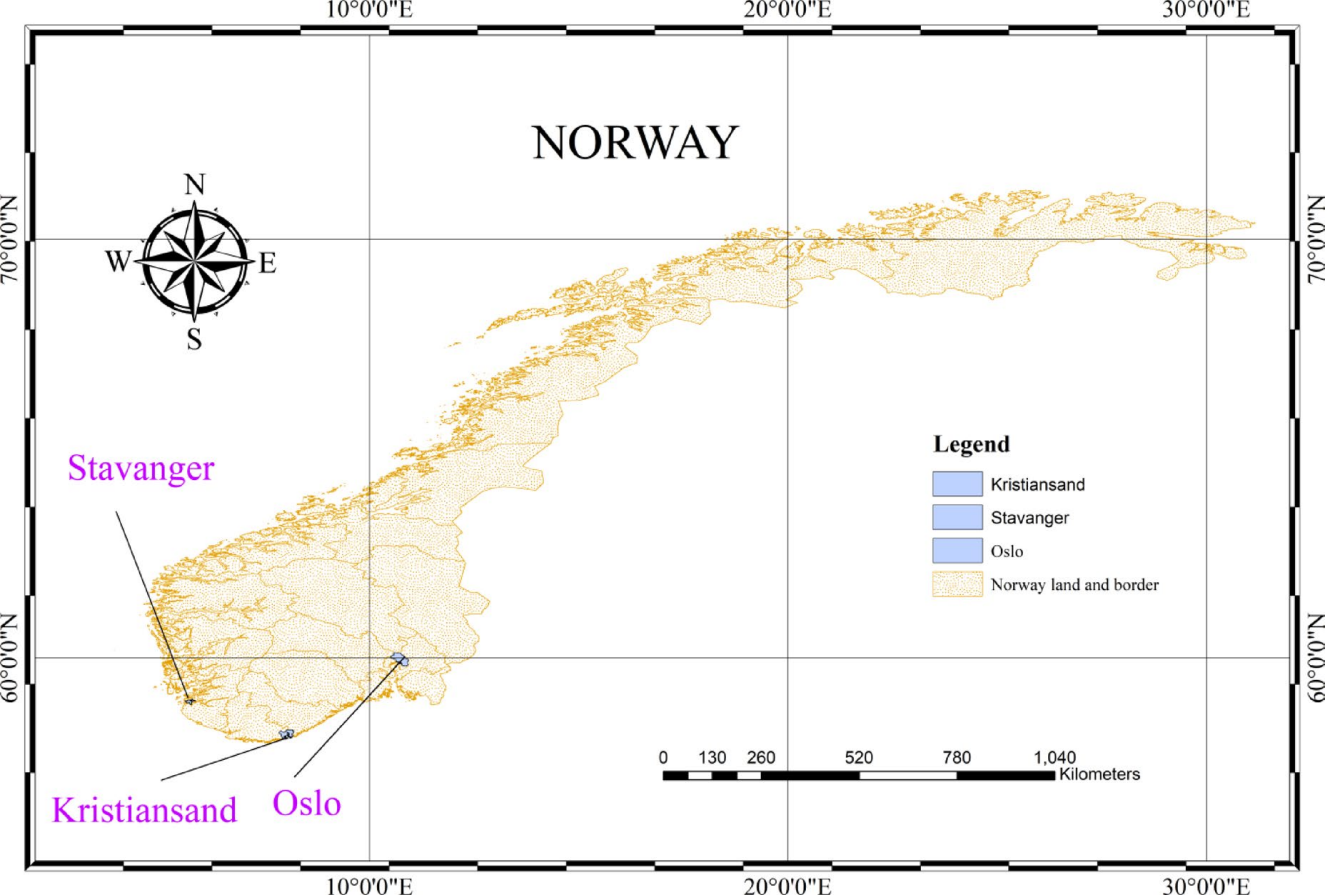
Study locations in southern Norway.. The map was generated using ArcMap (version 10.8) from ArcGIS Desktop (https://desktop.arcgis.com).

In this study, hourly PV output and temperature records were first obtained and then aggregated to a daily scale for forecasting. Hourly PV output contains structurally zero values during nighttime; if retained, these long zero sequences can dominate error metrics and encourage trivial predictions dominated by nocturnal conditions rather than the physically informative daylight signal. To focus on daytime generation, nighttime hours were excluded and the daily PV target was computed as the total PV power generated during a representative 7-hour daylight window (selected to consistently capture peak sun hours for the region and to represent daytime generation consistently across the study period). Daily mean air temperature was used as the meteorological predictor. This aggregation inevitably reduces intra-day variability; therefore, sub-daily (hourly) forecasting and uncertainty quantification are recommended directions for future work. Table 1 represents statistics of data used in this study (Fig. 2).

**Table 1:** Site characteristics and summary statistics of the dataset.

Station	Latitude (°*N*)	Longitude (°E)	Elevation (m)	Slope (°)	Azimuth (°)	Range (daily)
Kristiansand	58.153	7.99	50	47	0	2020–2023
Stavanger	58.971	5.725	37	46	-1	2020–2023
Oslo	59.898	10.737	0	49	-5	2020–2023

	Temperature (mean, °C**)**	PV power (mean)	Missing data	Std. deviation		
Kristiansand	8.18	3223	0	2144		
Stavanger	7.87	2784	0	2010		
Oslo	7.05	2865	0	2062		

### Model structure

**Fig. 3:**
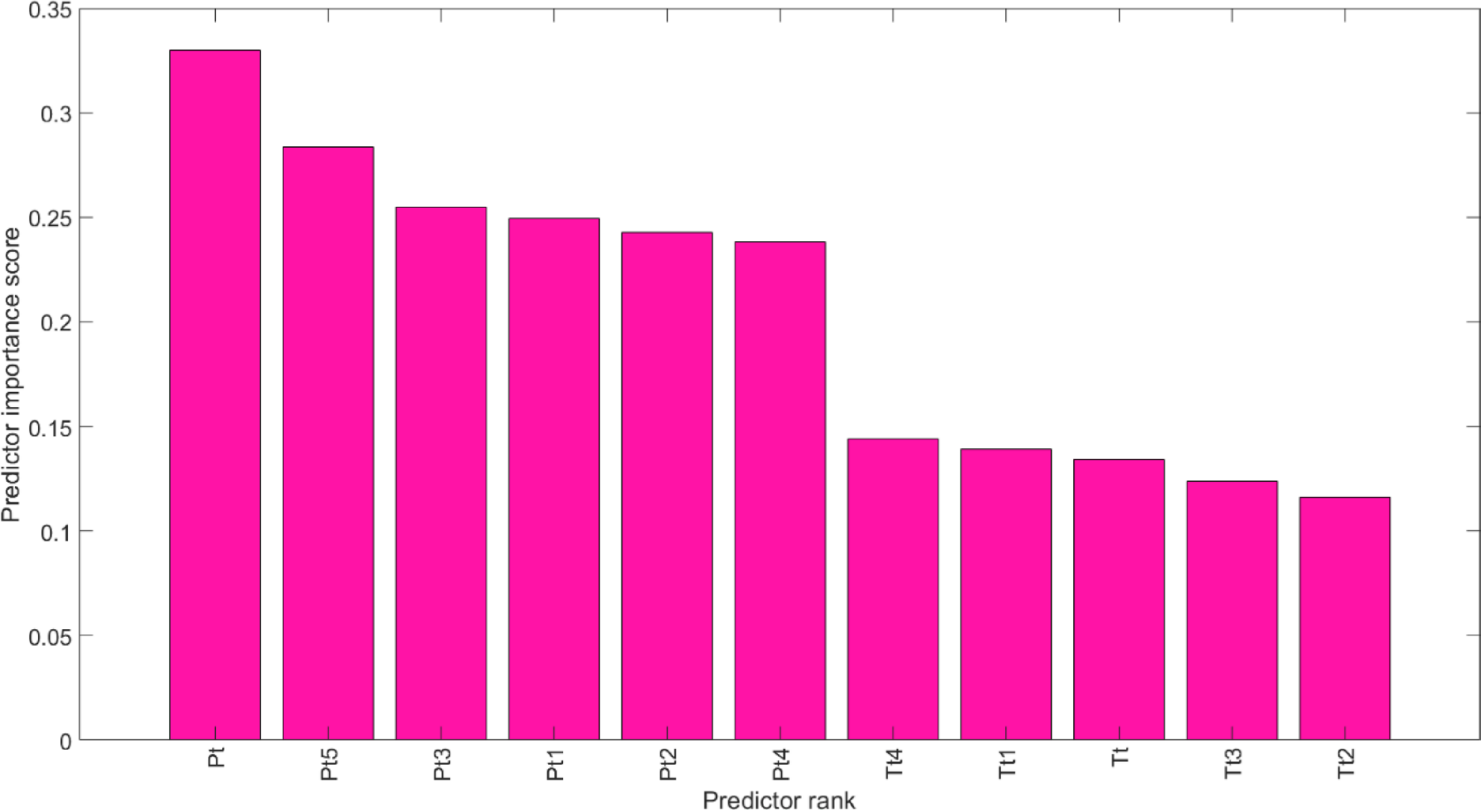
Feature importance results from the MRMR analysis. (Pt = PV target value on day t; Pt5 = PV value lagged by 5 days; Tt4 = temperature value lagged by 4 days; etc.).

Using different model structures in analyses may either increase or decrease model performance. Numerous studies and applications related to this topic can be found in the literature. Researchers often prefer feature selection methods grounded in statistical or stochastic process theory, and MRMR is one such technique. The Minimum Redundancy Maximum Relevance (MRMR) method is an effective feature selection technique designed to identify the optimal subset of input features for predicting an output. Its primary goal is to select features that are highly relevant to the output while maintaining minimum redundancy among themselves^76^. By prioritizing crucial, uncorrelated features, MRMR can enhance machine learning model accuracy and significantly reduce the risk of overfitting based on a greedy algorithm and a relevance-redundancy measure, making it particularly effective for high-dimensional datasets. In this study, the MRMR method was used to define the input structure for our models, based on its preference in the literature for yielding significant results^77^. For more details on the method, see Ding and Peng^76^. Through the MRMR, lagged input structures from PV output and air temperature were constructed. The significance levels and lagged values of temperature and PV data at different time intervals. Figure 3 shows the feature importance ranking from the MRMR analysis, and the resulting input combinations are summarized in Table 2.

**Table 2:** Input variables for each model configuration (M01–M04) determined by the MRMR feature selection.

	Inputs				Output
M01				P_t−5_	P_t_
M02			P_t−5_	P_t−3_	P_t_
M03		P_t−5_	P_t−3_	T_t−4_	P_t_
M04	P_t−5_	P_t−3_	T_t−4_	T_t−1_	P_t_

where P; daily total PV power, T; daily average air temperature (°C).

### Support vector machine (SVM)

Support Vector Machines (SVMs) and their regression variant SVR rely on kernel functions to map nonlinear relationships in complex datasets. The support vector machine (SVM) is a classifier that belongs to the kernel approaches in machine learning. This learning system is employed to classify and predict the data fitness function, aiming to minimize mistakes in data categorization or the fitness function itself. To advance these methods not only the optimization kernel architectures or refining hyperparameters but also tailoring implementations for particular application contexts were used^78–81^. Wang et al.^82^ demonstrated that the Gaussian kernel’s spread parameter (γ) must fall within a specific range to ensure the model achieve optimal generalization. SVM is extensively utilized for both regression and classification problems. Owing to its adaptability and efficacy it is positioned as a premier method in machine learning. For practical applications, Kusuma and Kudus^83^ describe SVR as a regression method designed to control overfitting and demonstrate its application on mortgage survival data using a linear kernel. Theoretical foundations connecting SVMs to probabilistic frameworks emerged through Wang et al.‘s^84^ work linking Gaussian kernel density estimation (GKDE) to kernel-based learning. Their analysis revealed that Gaussian-kernel SVMs operate as probabilistic classifiers, thereby providing Bayesian justification for the algorithm’s empirical success.

.

The mathematical formulation of SVM is presented in Eq. (1), defining the relationship between input and output variables as follows:

1 $$\:f\left(x\right)\:=\:\left(w,\varphi\:\left(x\right)\right)+b$$

where $\:\varphi\:\left(x\right)$ denotes a high dimensional feature space, w represents weight vectore, and b referred as the bias term. For implementation details and an extended reference trail used in closely related hybrid SVM studies the readers can refer to previous studies of Oruc et al.^38^ and Oruc et al.^85^ and for foundational SVR/SVM theory and formulation, cite standard SVR/SVM sources.

### Optimization methods for hybrid models

In this study, SVM algorithm was used to predict PV. Optimization methods, which are ABC, GWO, GA, and PSO, were used to enhance model performances. Initially, algorithm learning was performed with 70%, this ratio is frequently preferred in the literature, and it has been reported by many researchers that it yields effective results, of the dataset obtained from the region. Besides, time series data was not shuffled during the analysis because the sequence of data is critically important in time series analysis. The data was separated into training and testing sets without altering this sequence. The split ratios were selected based on proportions commonly found in the literature^73,77,85^. No random shuffling was applied, in order to simulate real forecasting conditions. Furthermore, preliminary steps or precautions against overfitting were taken using performance metrics like PI. Then, the optimization techniques mentioned above were used to further improve the performance of these algorithms. The results obtained from these models were evaluated according to performance evaluation criteria, RMSE, r, PI, KGE and NSE. All models were trained on the training set, and their performance was evaluated on the test set as described below.

#### Artificial bee colony algorithm (ABC)

Karaboga^86^ modeled the Artificial Bee Colony (ABC) algorithm based on honeybee foraging behavior. The algorithm divides the colony into three functional groups which exhibit distinct search behaviors. Employed bees exploit known food sources and communicate their quality through waggle dances at the hive. Onlooker bees observe these dances, evaluate potential sources based on profitability, and focus search efforts on those promising locations. Scout bees are responsible for exploring search space randomly and identifying new sources when existing sites are depleted^87–89^.

The algorithm maintains a balance between exploration and exploitation through dynamic role transitions. When a particular food source fail to show improvement over successive iterations, the employed bee assigned that location abandons it and transitions into a scout bee. This conversion starts random exploration for new opportunities^90^. This abandonment mechanism prevents the algorithm from being trapped in suboptimal solutions. The scouts introduce diversity and novelty into the search process, employed and onlooker bees refine and extract benefit from known solutions. Mathematical details and implementation procedures provided in Karaboga^86^, Li et al.^88^, and Vitorino et al.^89^.

#### Grey Wolf optimizer (GWO)

Mirjalili et al.^91^ introduced the Grey Wolf Optimizer (GWO) by modeling the hunting dynamics observed in grey wolf packs. The algorithm transforms this behavior into a computational optimization framework. Grey wolves hunt through a hierarchical system of coordinated roles that includes tracking prey, encircling, and attacking. This is a strategy that translates effectively into computational search patterns^92,93^. The straightforward linear structure of the algorithm simplifies implementation while maintaining performance and has produced successful results in many fields, as mentioned by numerous researchers^94^.

GWO establishes candidate solutions based on the observed wolf pack hierarchy. The alpha (α) represents the current best solution and positions itself as the leader of the search process. Beta (β) and delta (δ) wolves correspond to the second and third-best solutions that guide exploration of potential regions. All remaining solutions function as omega (ω) wolves that represents the pack’s lowest tier that explores the broader search space^91,95^. This hierarchical structure drives the optimization mechanism forward. Alphas direct the hunt, betas and deltas refine the search direction and omegas ensure diversity in exploration.

Mathematically, the three hunting phases translate into iterative position adjustments. These are namely, tracking, encircling, and attacking phases which are guided by the alpha, beta, and delta solutions. Each iteration recalculates and refines wolf positions based on their distance from these leaders. The process progressively tightening the search around optimal regions until the algorithm reaches convergence, that is two criteria are determined in the GWO process: (1) catching the prey (reaching the best solution) and (2) reaching the maximum number of iterations^96^. Details governing the process can be found in Mirjalili et al.^91^.

#### Genetic algorithm (GA)

Holland introduced the Genetic Algorithm (GA) in 1960 and refined it over the following two decades. The algorithm translates Darwin’s evolutionary principles into computational optimization methods. GA maintains a population of potential solutions that compete, recombine, and randomly mutate across iterative generations. This process directly parallels biological evolution where advantageous characteristics persist through populations while disadvantageous ones disappear^44^ and^97^. Industrial applications have validated the effectiveness of the algorithm across diverse application domains, ranging from parameter optimization to complex scheduling problems^46,47,98,99^.

The fundamental strength of the algorithm lies in its gradient-free search mechanism. This feature enables it to handle both continuous and discrete optimization challenges without requiring gradient calculations. Such a requirement constrains many traditional optimization methods^44^ and^97^. Bras et al.^100^ documented GA’s flexibility through applications extending from linguistic analysis to fuzzy network tuning. Their work demonstrates how the same evolutionary mechanisms, namely crossover, mutation, and selection, can be adapted to different problem domains^100,101^.

The algorithm proceeds through three core stages. First it initializes a random population, second applies genetic operators through probabilistic rather than deterministic process, and third evaluates solution quality until convergence criteria trigger termination^101,102^. When solutions fail to meet established quality thresholds, the algorithm iterates through another generational cycle. This iteration systematically refines and enhances the population through selection pressure and genetic variation^101,103^.

These steps are:

1. Crossover (stochastic): part of two solutions “is swapped” to produce new ones.
2. Mutation (stochastic): part of a new solution “is flipped” to generate a new one and prevent it from converging into local optima.
3. Selection: the new solutions are evaluated according to the objective function, and the best candidates are selected.

In certain instances, such as a high mutation rate that could lead to the loss of good solutions, the elitism operator is employed to guarantee that the optimal solutions are transferred to the next generation without modification, ensuring that the best candidates are maintained within the solution set^100,103^.

#### Particle swarm optimization (PSO)

Kennedy and Eberhart introduced particle swarm optimization (PSO) in 1995. This algorithm conceptually inspires from coordinated and collective animal behaviors observed in natural system such as schools of fish navigating currents and bird flocks foraging collectively. Unlike genetic algorithms and other comparable evolutionary methods, PSO achieves more rapid convergence while requiring fewer computational resources. This advantage becomes particularly evident when addressing nonlinear optimization problems and impact modeling success^104,105^. The algorithm treats each potential solution as a particle moving through complex search spaces and is to use information of the current position X and velocity V of particles^106^.

Initially, particles are positioned randomly across the solution space. As the algorithm progresses through successive iterations, each particle adjusts its trajectory based on two guiding influences. These are the particle’s best position discovered so far and the globally best position identified by any particle of the entire swarm. This dual-component memory system drives particles toward potential solution regions while simultaneously maintaining exploration capability^107–111^. The search continues until particles converge on a shared optimal location. This convergence signals that the algorithm has identified the best available solution within the search space^112–114^.

### Performance metrics

The correlation coefficient (r), root mean square error (RMSE), Nash-Sutcliffe efficiency (NSE), Kling-Gupta efficiency (KGE), and Performance Index (PI), were employed key statistical indicators to assess model performance as specified in Equations (2) through (6). (2), (3), (4), (5), and (6) NSE and KGE are goodness-of-fit measures that attain 1 for perfect prediction. For NSE, a value of 0 indicates performance equivalent to the mean-prediction baseline, whereas KGE uses a benchmark threshold of approximately − 0.41 for comparable interpretation^115^.

2 $$r = \frac{{\sum {_{{i = 1}}^{N} } \left. {\kern-0.15em \left( x \right. \kern-0.15em _{{pi}} - \bar{x}_{p} } \right)\left( {x_{{oi}} - \bar{x}_{o} } \right)}}{{\sqrt {\sum {_{{i = 1}}^{N} } \left. { \kern-0.15em \left( x \right. \kern-0.15em _{{pi}} - \bar{x}_{p} } \right)^{2} } *\sqrt {\sum {_{{i = 1}}^{N} } \left( {x_{{oi}} - \bar{x}_{o} } \right)^{2} } }}$$

3 $$\:RMSE\:=\:\sqrt{\frac{1}{N}{\sum\:}_{i=1}^{N}{\left({x}_{oi}-{x}_{pi}\right)}^{2}}$$

4 $$NSE = 1 - \left[ {\frac{{\sum {_{{i = 1}}^{N} } \left( {x_{{oi}} - x_{{pi}} } \right)^{2} }}{{\sum {_{{i = 1}}^{N} } \left( {x_{{oi}} - \bar{x}_{o} } \right)^{2} }}} \right]$$

5 $$\:KGE\:=\:1\:-\:\sqrt{{\left(r-1\right)}^{2}\:+{\:\left(a-1\right)}^{2}\:+\:{\left(\beta\:-1\right)}^{2}}$$

$$\:\beta\:\:=\:\frac{{x}_{p}}{{x}_{o}},\:\:a\:=\:\frac{{\sigma\:}_{{x}_{p}}}{{\sigma\:}_{{x}_{o}}}$$

6 $$PI = \frac{{\frac{{RMSE}}{{\left| {\bar{x}_{p} } \right|}}}}{{1 + r}}$$

## Results

In this study, total daily PV power generation data and daily average temperature values obtained from Kristiansand, Stavanger, and Oslo, which are in the southern region of Norway, were used to investigate the performance of forecasting models that were developed through combination of machine learning and optimization techniques. A total of 70% of the data was employed for training, while the remaining 30% was reserved for testing. Support Vector Machines (SVM) were selected as the machine learning algorithm, whereas optimization techniques included the Artificial Bee Colony (ABC), Grey Wolf Optimizer (GWO), Genetic Algorithm (GA), and Particle Swarm Optimization (PSO) due to one of the primary objectives of the study which is to enhance model performance by hybridizing. Furthermore, in order to examine the effect of model input variables on prediction performance, the MRMR (Minimum Redundancy Maximum Relevance) method was applied to construct four different input structures. The results of all analyses are presented in Table 3. Furthermore, it should be noted that these results and rankings (e.g., PSO-M04 being best) are specific to the data and sites analyzed; generalizing beyond this case study should be done with caution.

According to the results presented in Table 3, the performance metrics of the four input structures analyzed with SVM in the Kristiansand region differ from one another. Among these, the lowest performance was observed in M01, while the strongest baseline (untuned) SVM results were obtained with M04 (*r* = 0.6469, NSE = 0.3587, KGE = 0.3141, PI = 0.2937, and RMSE = 0.8004). The analysis of model structures shows that M01 was built using one input variable (Pt-5), while M04 was built using four input variables (Pt-5, Pt-3, Tt-4, and Tt-1). While both models used Pt as the target variable, integrating power data with temperature improves daily forecasting precision based on the results. Evidence from the analysis also reveals the success of MRMR-based input selection process in identifying superior input combinations. MRMR-based input selection process when coupled with hybridization of SVM with metaheuristic optimizers, also a consistent improvement was obtained in performance metrics (Table 3). Therefore, results also validate the value of hybridizing machine learning with optimization techniques compared to standalone configurations. However, the differences were marginal, which makes it challenging to identify a single superior optimizer for this region. Despite this, a closer look at the performance metrics revealed that SVM-PSO-M04 delivered the best test results for the Kristiansand region (*r* = 0.7707, NSE = 0.5748, KGE = 0.7092, PI = 0.2964, and RMSE = 0.6513). Among the tested models it can be concluded that metaheuristic hyperparameter tuning can enhance SVM performance for forecasting in this study.

Compared to the analysis without any optimization technique (SVM-M04), the performance metrics were improved. Additionally, when the results of the other optimization methods were evaluated, the M04 model consistently demonstrated success across all cases. This finding further validates the effectiveness of the MRMR method in determining the model input structure for this region; while these results should be interpreted as site- and dataset-specific.

**Table 3:** Results of all model configurations and optimizers for each region.

Kristiansand																	
SVM-ABC						SVM-GWO						SVM-GA					
	r	NSE	KGE	PI	RMSE		r	NSE	KGE	PI	RMSE		r	NSE	KGE	PI	RMSE
M01	0.6870	0.4465	0.5765	0.3549	0.7431	M01	0.6870	0.4443	0.5673	0.3556	0.7446	M01	0.6886	0.4506	0.5696	0.3533	0.7404
M02	0.7442	0.5409	0.6534	0.3126	0.6768	M02	0.7661	0.5610	0.7095	0.3019	0.6618	M02	0.7655	0.5610	0.7013	0.3020	0.6618
M03	0.7689	0.5706	0.7112	0.2981	0.6545	M03	0.7688	0.5714	0.7062	0.2979	0.6539	M03	0.7574	0.5555	0.6920	0.3053	0.6660
**M04**	**0.7712**	**0.5739**	**0.7147**	**0.2966**	**0.6520**	**M04**	**0.7706**	**0.5730**	**0.7147**	**0.2970**	**0.6527**	**M04**	**0.7677**	**0.5689**	**0.7024**	**0.2989**	**0.6559**
SVM-PSO						SVM											
	r	NSE	KGE	PI	RMSE		r	NSE	KGE	PI	RMSE						
M01	0.6870	0.4482	0.5482	0.3544	0.7420	M01	0.5636	0.1803	0.0878	0.3497	0.9049						
M02	0.7673	0.5659	0.7054	0.3000	0.6581	M02	0.6502	0.3144	0.2433	0.3030	0.8276						
M03	0.7686	0.5719	0.7034	0.2977	0.6536	M03	0.6541	0.3471	0.2886	0.2950	0.8076						
**M04**	**0.7707**	**0.5748**	**0.7092**	**0.2964**	**0.6513**	**M04**	**0.6469**	**0.3587**	**0.3141**	**0.2937**	**0.8004**						
*Stavanger*																	
SVM-ABC						SVM-GWO						SVM-GA					
	r	NSE	KGE	PI	RMSE		r	NSE	KGE	PI	RMSE		r	NSE	KGE	PI	RMSE
M01	0.6039	0.3622	0.4504	0.4012	0.7977	M01	0.6039	0.3607	0.4693	0.4017	0.7987	M01	0.6039	0.3613	0.4626	0.4015	0.7983
M02	0.6693	0.4456	0.5357	0.3594	0.7437	M02	0.6693	0.4455	0.5317	0.3595	0.7438	M02	0.6122	0.3954	0.5012	0.3645	0.7741
**M03**	**0.6799**	**0.4617**	**0.5500**	**0.3519**	**0.7328**	M03	0.6799	0.4602	0.5745	0.3524	0.7339	M03	0.6799	0.4603	0.5715	0.3524	0.7338
M04	0.6796	0.4614	0.5510	0.3521	0.7331	**M04**	**0.6796**	**0.4613**	**0.5549**	**0.3521**	**0.7331**	**M04**	**0.6797**	**0.4616**	**0.5522**	**0.3520**	**0.7329**
SVM-PSO						SVM											
	r	NSE	KGE	PI	RMSE		r	NSE	KGE	PI	RMSE						
M01	0.6039	0.3622	0.4504	0.4012	0.7977	M01	0.5355	0.1647	0.0697	0.4066	0.9135						
M02	0.6693	0.4456	0.5347	0.3594	0.7437	M02	0.6163	0.2841	0.2153	0.3576	0.8457						
**M03**	**0.6798**	**0.4619**	**0.5469**	**0.3518**	**0.7327**	M03	0.6221	0.3010	0.2421	0.3521	0.8356						
M04	0.6796	0.4614	0.5512	0.3521	0.7330	**M04**	**0.6091**	**0.3018**	**0.2543**	**0.3548**	**0.8352**						
*Oslo*																	
SVM-ABC						SVM-GWO						SVM-GA					
	r	NSE	KGE	PI	RMSE		r	NSE	KGE	PI	RMSE		r	NSE	KGE	PI	RMSE
M01	0.5883	0.3319	0.4460	0.4300	0.8164	M01	0.5883	0.3266	0.4365	0.4317	0.8197	M01	0.5883	0.3308	0.4311	0.4304	0.8171
M02	0.6575	0.4171	0.5684	0.3849	0.7626	M02	0.6542	0.4156	0.5331	0.3862	0.7636	M02	0.6542	0.4145	0.5450	0.3865	0.7643
**M03**	**0.6721**	**0.4352**	**0.5988**	**0.3756**	**0.7507**	M03	0.6628	0.4235	0.5826	0.3815	0.7584	**M03**	**0.6761**	**0.4342**	**0.6118**	**0.3750**	**0.7513**
M04	0.6673	0.4345	0.5195	0.3769	0.7511	**M04**	**0.6671**	**0.4339**	**0.5318**	**0.3771**	**0.7515**	M04	0.6645	0.4290	0.5679	0.3794	0.7548
SVM-PSO						SVM											
	r	NSE	KGE	PI	RMSE		r	NSE	KGE	PI	RMSE						
M01	0.5883	0.3329	0.4203	0.4297	0.8159	M01	0.5193	0.1572	0.0577	0.3875	0.9176						
M02	0.6624	0.4209	0.5762	0.3825	0.7601	M02	0.5975	0.2692	0.1960	0.3432	0.8545						
M03	0.6622	0.4272	0.5501	0.3805	0.7559	M03	0.6044	0.2938	0.2300	0.3359	0.8400						
**M04**	**0.6861**	**0.4470**	**0.6252**	**0.3686**	**0.7428**	**M04**	**0.5994**	**0.3043**	**0.2518**	**0.3344**	**0.8336**						

Stavanger is another station within the study area where analyses were conducted. At this station, the same methods as in the previous case were applied, and the results are presented in Table 3. In the analyses performed solely with SVM, the M04 model achieved the highest scores among the four input structures (*r* = 0.6091, NSE = 0.3018, KGE = 0.2543, PI = 0.3548, and RMSE = 0.8352). Similar to the previous station, the input structure of M04 also demonstrated effective performance in this part of the analysis. For this station as well, optimization techniques were utilized to improve model performance. In the analyses involving ABC, GWO, GA, and PSO, performance metrics generally improved, with the results being very close to each other. Therefore, it was difficult to identify a single best-performing algorithm for the Stavanger station also. A primary takeaway of the analyses can be the overall performance among the optimization algorithms which produced comparable results. Another key observation is while the highest score metrics were obtained from M03 with the SVM-ABC and SVM-PSO techniques, all other methods achieved their highest scores using M04. These results suggest that the effectiveness of model input structures may vary upon regional environmental characteristics and they are sensitive to those characteristics. An additional finding is that using an optimal number of input variables with expanded data diversity which means balancing input complexity with representativeness, can achieve higher predictive precision.

Another station from which data were collected within the study area is Oslo. All of these stations are located in the southern region of Norway, where sunshine duration is considerably higher compared to other regions located in the country. The analysis results obtained from this station are presented in Table 3. As in the other stations, analyses were first performed using only SVM, and optimization techniques were subsequently applied to improve the results. Overall, the analyses incorporating optimization techniques improved the performance metrics of all models. In the analyses performed solely with SVM, the highest scores were obtained with M04 (*r* = 0.5994, NSE = 0.3043, KGE = 0.2518, PI = 0.3344, and RMSE = 0.8336). Among the optimization-based analyses, SVM-PSO-M04 achieved the highest test scores (*r* = 0.6861, NSE = 0.4470, KGE = 0.6252, PI = 0.3686, and RMSE = 0.7428), although differences among the top optimizers were modest. Unlike the Stavanger station, the best-performing input structure at this station was obtained with M04. Therefore, it can be concluded that while determining model input structures, not only PV power data but also temperature information can be beneficial for daily PV forecasting.

In general, across all regions, in this dataset, the strongest results were most often obtained with M04, while the input structure M01 exhibited the lowest performance metrics. All optimization techniques improved the performance of the models relative to the untuned SVM baseline. However, because performance differences among optimizers were relatively small, PSO should be interpreted as yielding the highest scores in this study rather than as universally superior.

**Fig. 4:**
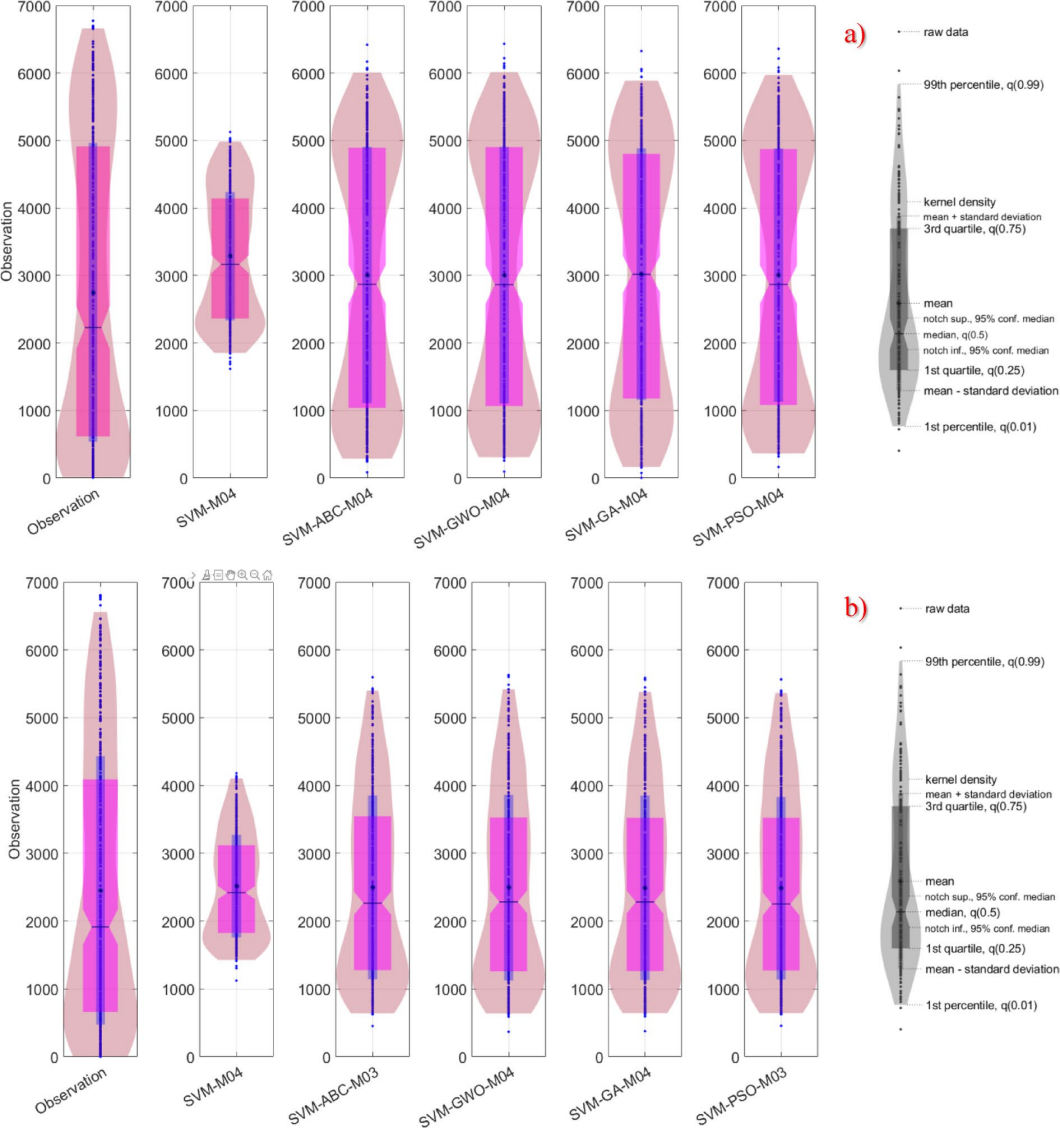

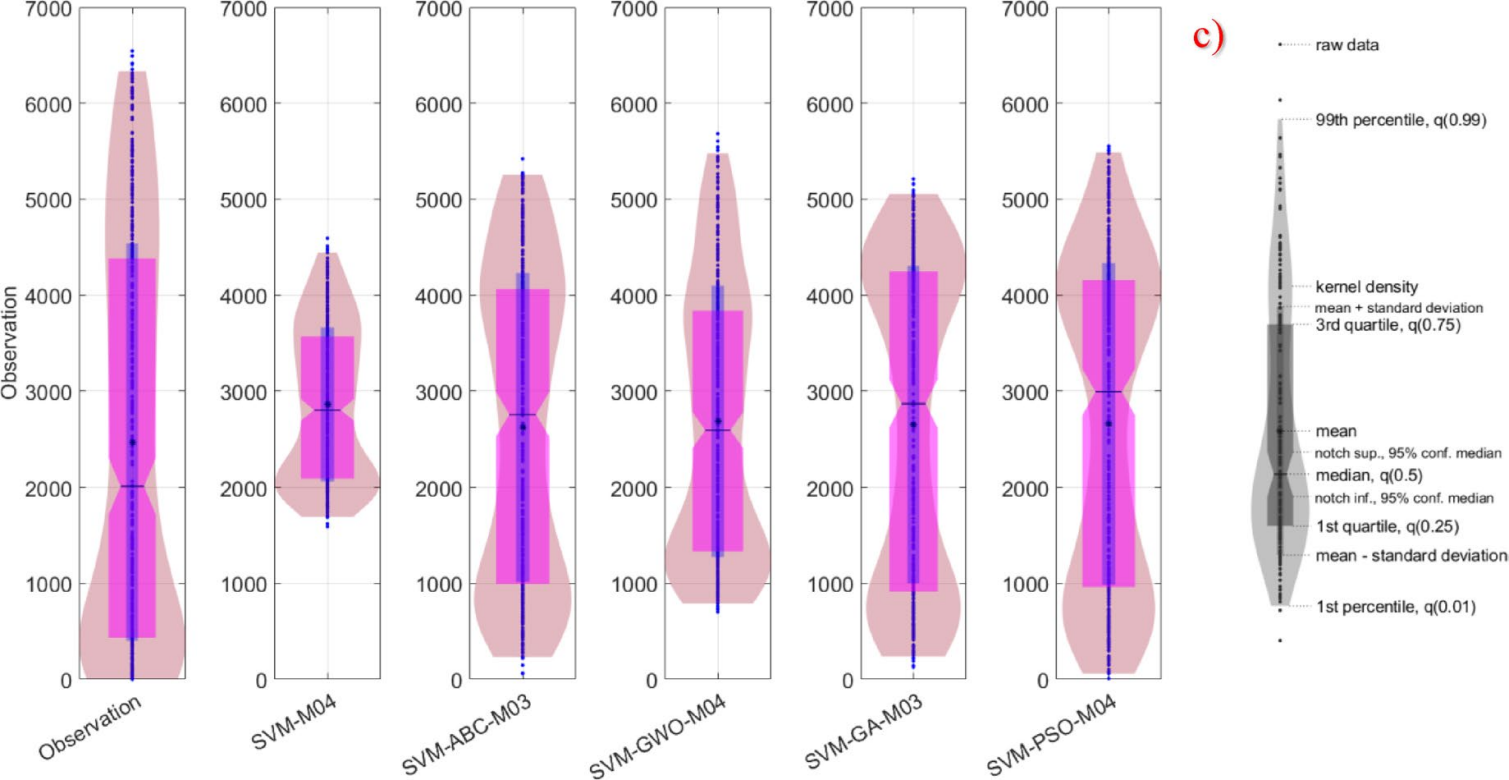
Violin diagram for all models in different stations on study area where *SVM-M04*; analysis of baseline SVM with M04 and, *SVM-ABC-M04*; analysis of SVM coupled ABC (hybridized SVM) with M04 etc. (**a**) Kristiansand (**b**) Stavanger (**c**) Oslo.

In this study, in addition to statistical calculations, visual comparison methods were also employed. For the three different stations within the study area, violin plots were generated and are presented in Fig. 4. These plots were constructed to enable the comparison of the best-performing models within each category. According to the results, the only model that did not visually align with the observed values for Kristiansand was SVM-M04. Almost all of the other plots displayed highly similar distributions. Therefore, the most critical aspects to consider in this context are measures such as the mean, extremes, and median.

Although it is rather difficult to distinguish between the models based on visual observations alone, an evaluation of kernel densities, the 3rd quartile, and median values revealed that the model most similar to the observed data was SVM-PSO-M04. In Stavanger, the best-performing model was identified as SVM-PSO-M03, while in Oslo, the most successful model was again SVM-PSO-M04. In determining these successful models, multiple statistical parameters from the violin plots were taken into consideration.

**Fig. 5:**
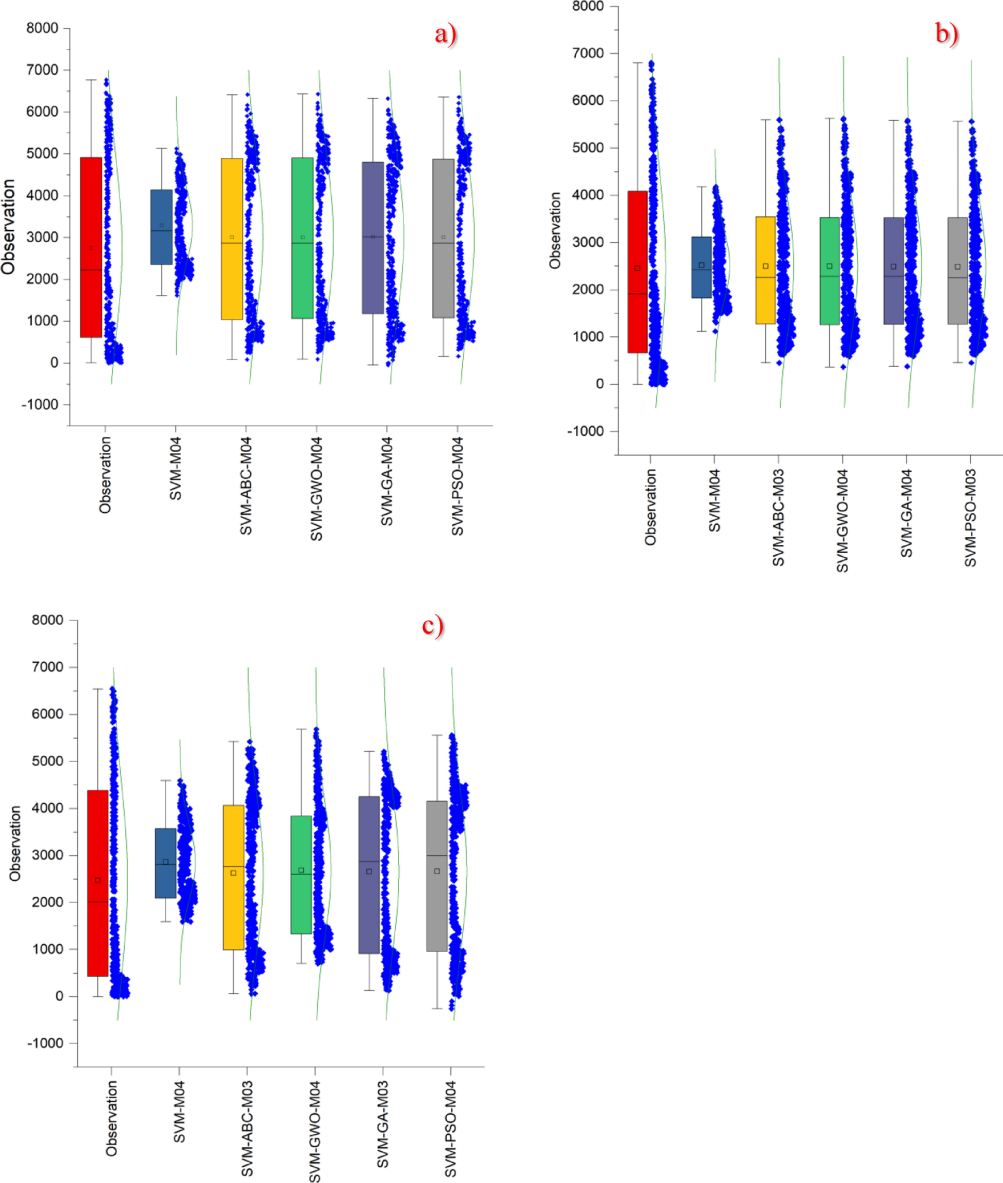
Box-normal diagram for all models in different stations on study area where *SVM-M04*; analysis of SVM with M04, *SVM-ABC-M04*; analysis of SVM and ABC with M04 etc. (**a**) Kristiansand (**b**) Stavanger (**c**) Oslo.

In Fig. 5, a box-normal plot is presented to enable a better comparison of the best-performing models. Upon detailed examination of this plot, it appears quite difficult to distinguish the differences between the observed values and the prediction models. This difficulty mainly arises from the fact that all optimization methods produced results that were very close to each other. While the prediction models in the Kristiansand and Stavanger regions yielded highly similar outcomes, a few of the prediction models in the Oslo region could be distinguished more easily. In this region, the SVM-ABC-M03 and SVM-GWO-M04 models visually resembled the observed values more closely than the others; however, in terms of statistical results, these two methods lagged behind. Thus, the visual outcomes and the statistical results did not fully align. In conclusion, when the performance metrics obtained from prediction models are very close to one another, the box-normal method should not be preferred for visual comparison.

**Fig. 6:**
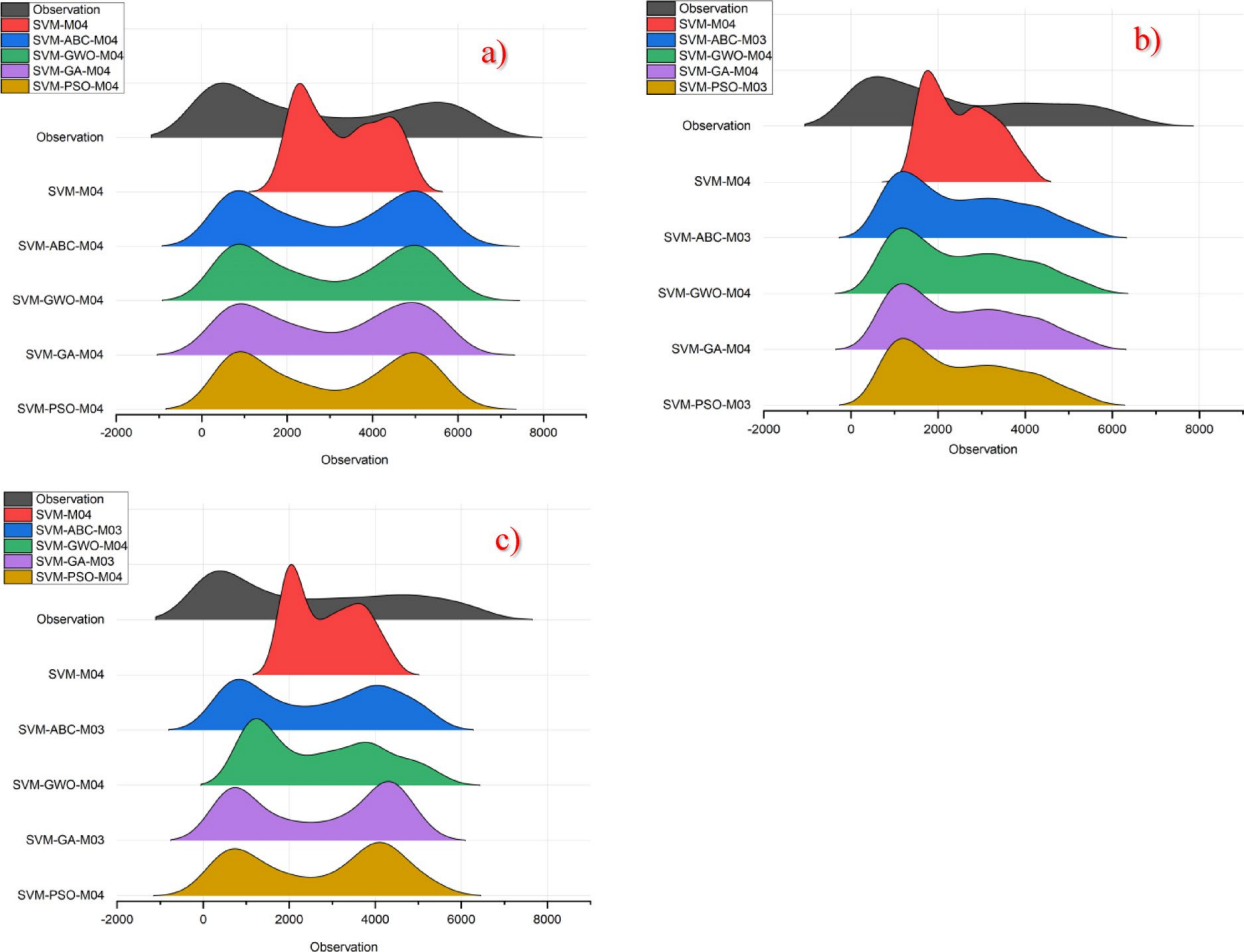
Ridge diagram for all models in different stations on study area where *SVM-M04*; analysis of SVM with M04, *SVM-ABC-M04*; analysis of SVM and ABC with M04 etc. (**a**) Kristiansand (**b**) Stavanger (**c**) Oslo.

The ridge plot, another visual comparison method, is also included in this study. Figure 6 shows the ridge plot for all models that were successful in their respective class. Upon examining this plot, a model that visually overlaps with the observation values in the Kristiansand and Oslo regions could not be identified. However, in the Stavanger region, the models were able to predict the peak that occurred in the initial values of the observation data. But this was also not effective in determining the most successful model. Consequently, this visual comparison method cannot be considered successful either. In conclusion, the ridge plot was also unsuccessful in determining the best model, that is, in comparing prediction models whose performance metrics were very close to each other.

**Fig. 7:**
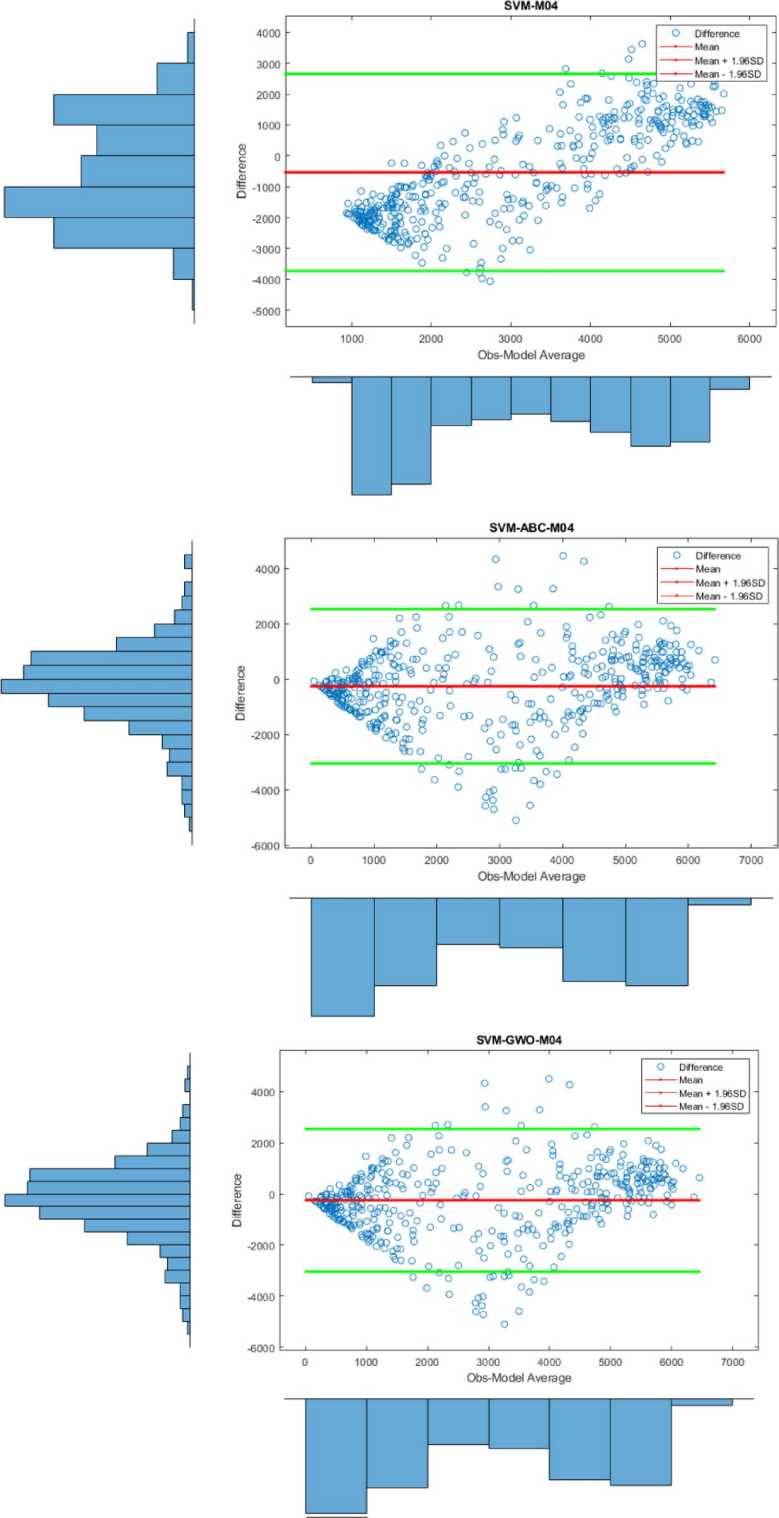

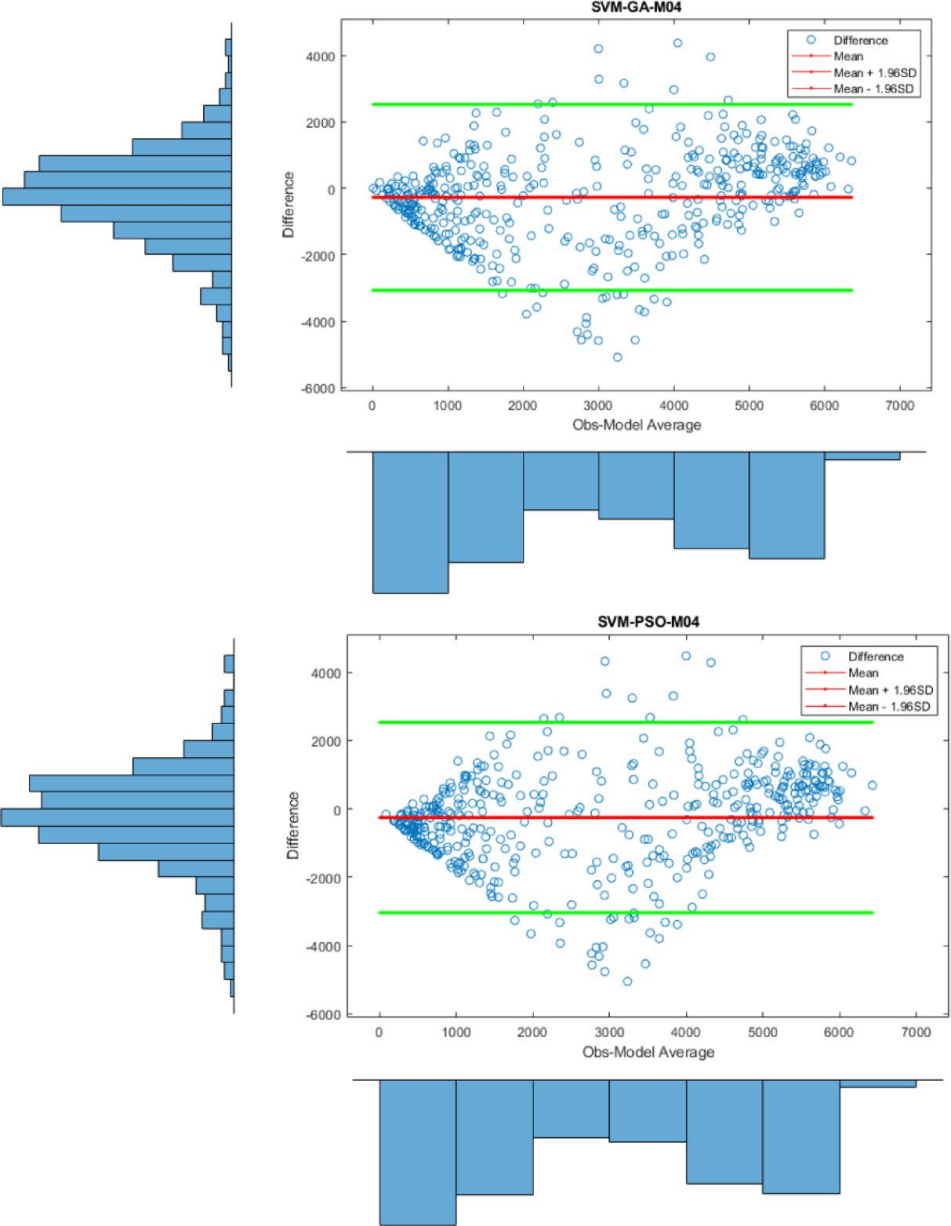
Bland-Altman diagram for all models in different stations on study area where *SVM-M04*; analysis of SVM with M04, *SVM-ABC-M04*; analysis of SVM and ABC with M04 etc. in Kristiansand.

**Fig. 8:**
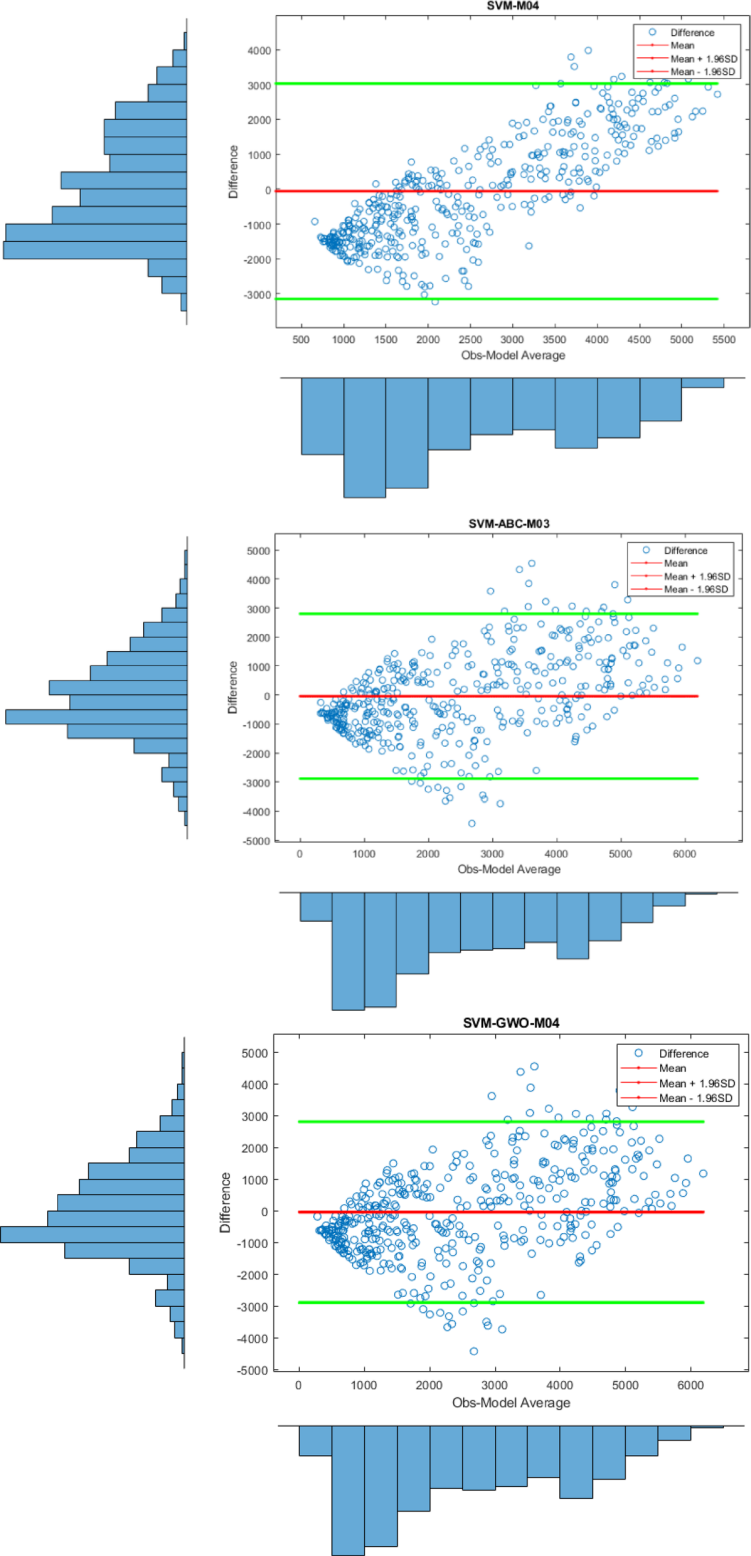

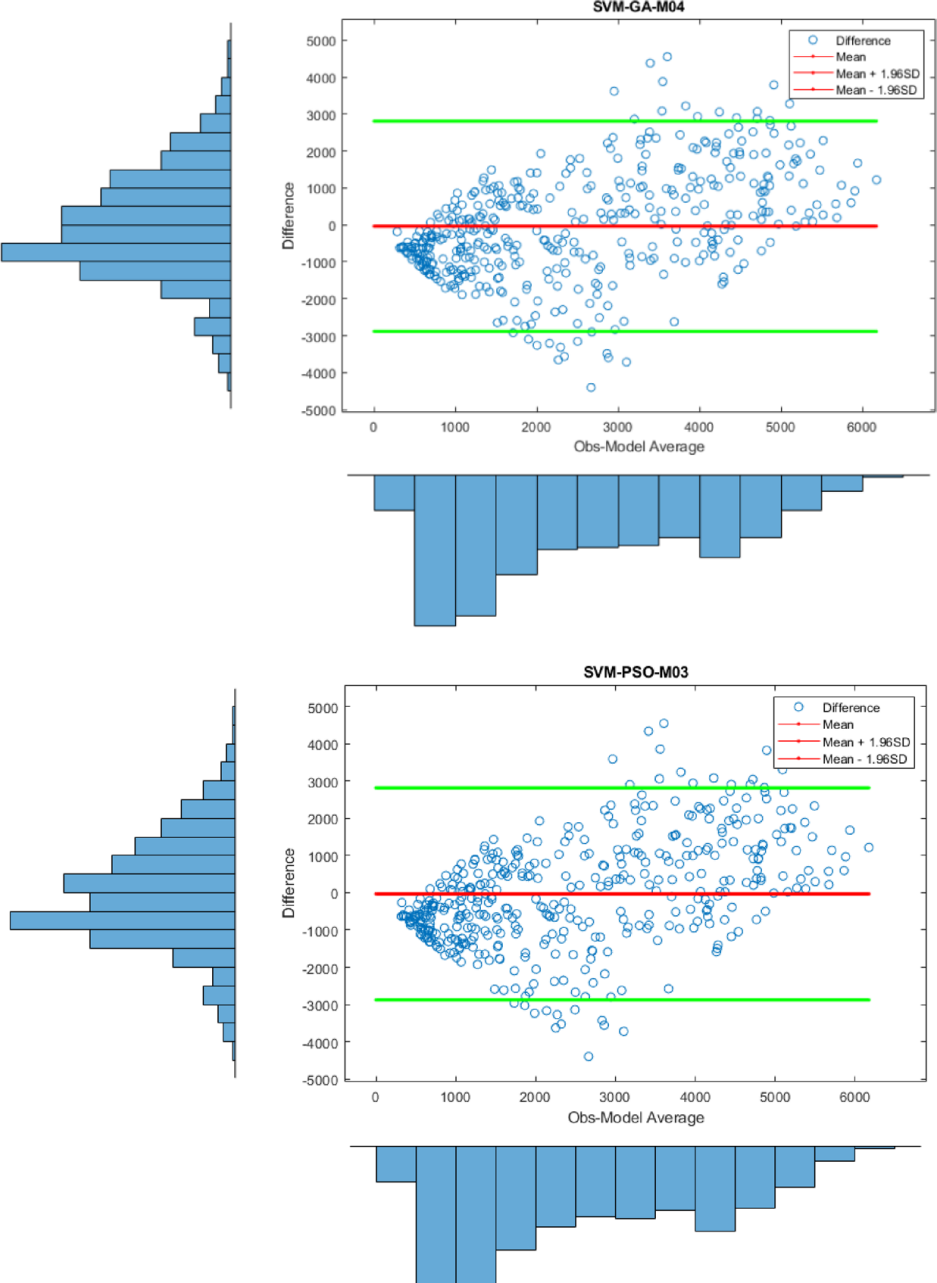
Bland-Altman diagram for all models in different stations on study area where *SVM-M04*; analysis of SVM with M04, *SVM-ABC-M04*; analysis of SVM and ABC with M04 etc. in Stavanger.

**Fig. 9:**
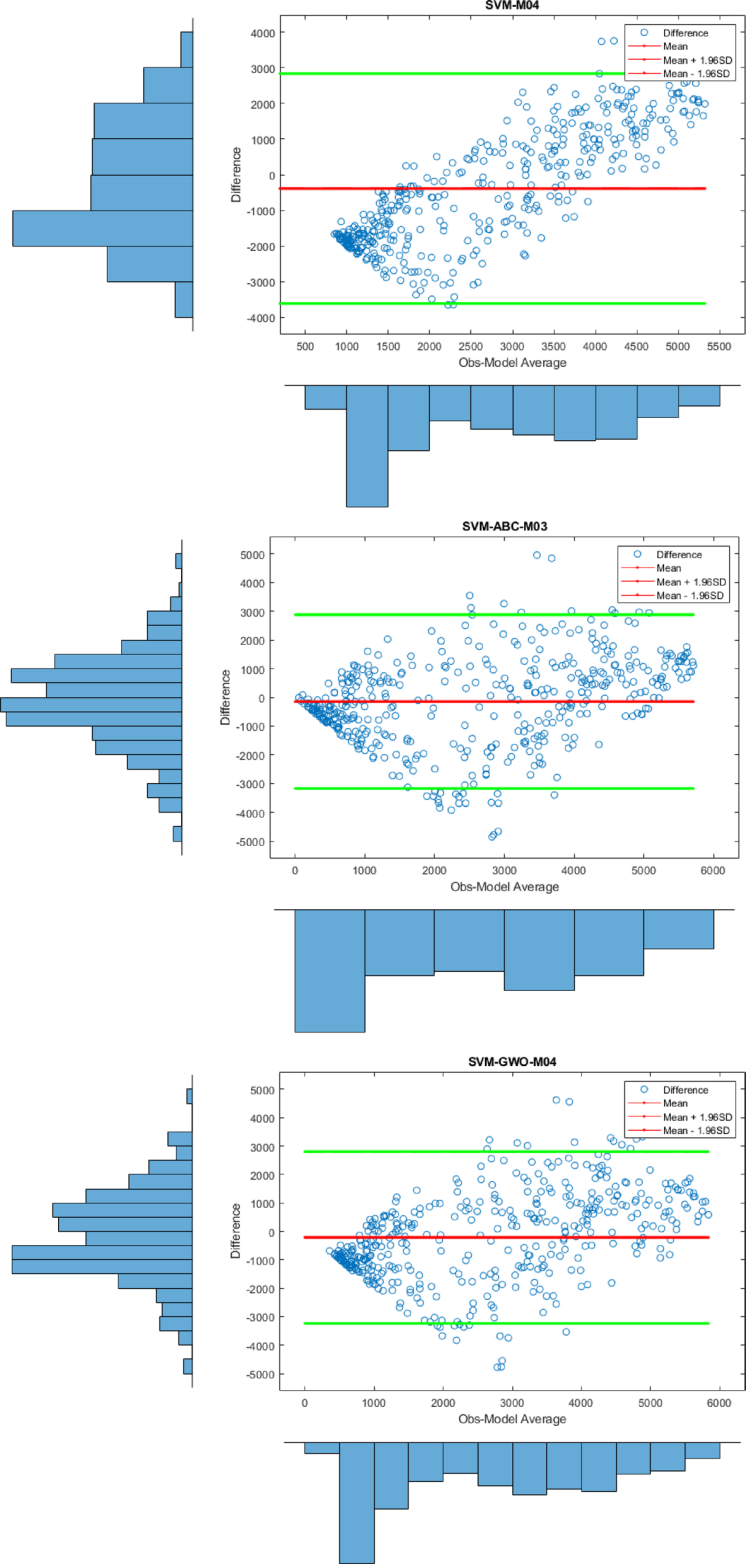

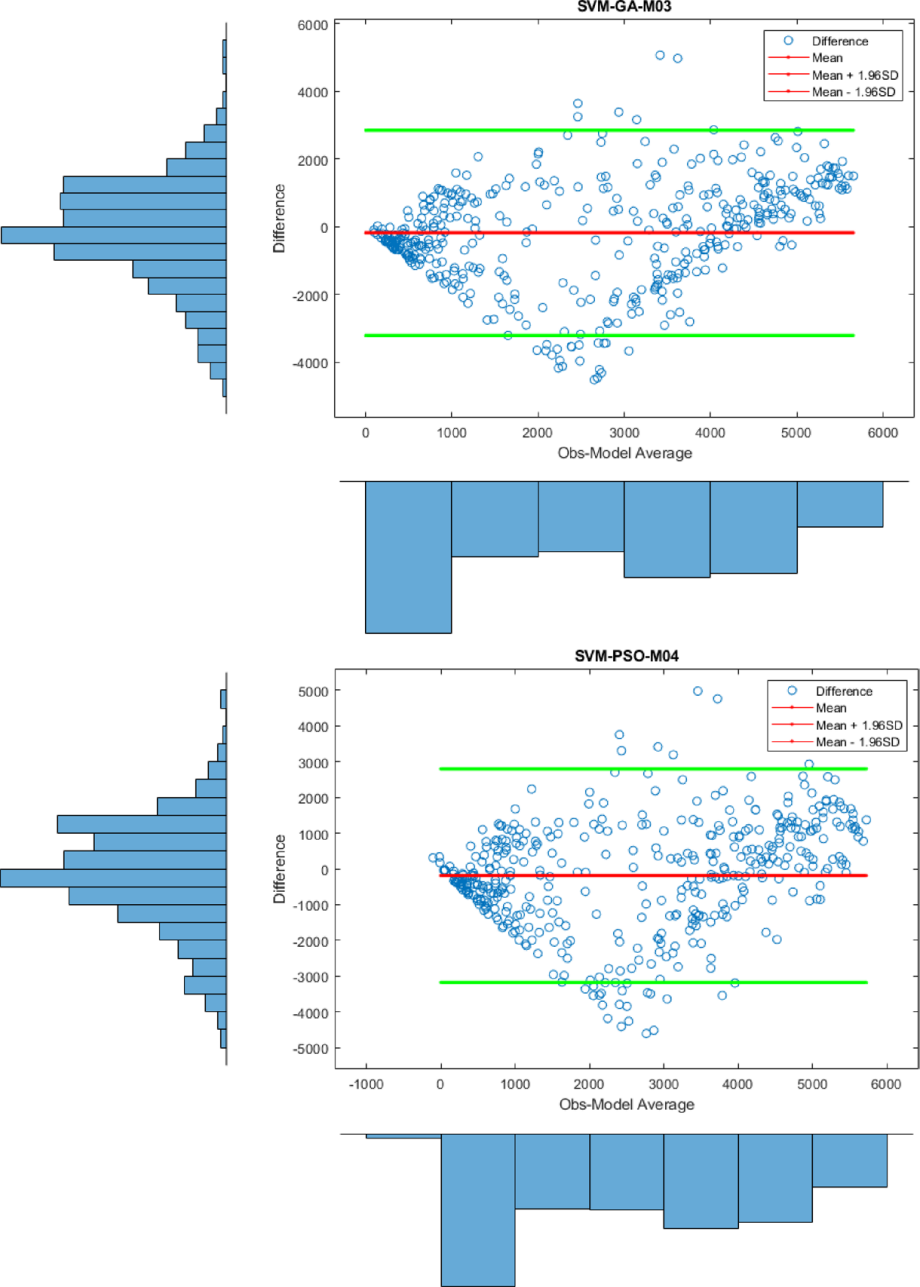
Bland-Altman diagram for all models in different stations on study area where *SVM-M04*; analysis of SVM with M04, *SVM-ABC-M04*; analysis of SVM and ABC with M04 etc. in Oslo.

Apart from the Violin plot, the visual comparison methods mentioned above did not yield exceptional results in distinguishing the most successful models. Therefore, to both increase the comparison comprehensiveness of the study and to enable a more qualified distinction of the results, Bland-Altman and Box plots were generated for all regions. All plots, specific to different regions, are shown in Figs. 7 and 8, and 9.

In Fig. 7, the differences between the prediction models based on the observed values at 95% limits of agreement level are shown. One of the most important results here is that the differences for the SVM-PSO-M04 model are notably clustered around the zero-line indicating little bias for the SVM-PSO-M04 model. Although the predicted values for the other models also concentrate around zero, the densest region was determined to be SVM-PSO-M04. Therefore, this is an indication that it is the most successful model in that region.

Figure 8, on the other hand, shows the differences between the prediction models for Stavanger at 95% limits of agreement level, based on the observed values. The key point is that the predicted values for all models failed to cluster around the zero line, meaning their predictions have larger deviations from observations. Therefore, this indicates that no conclusive results were found for Stavanger. However, when looking at the SVM-PSO-M03 model, the predicted values are clustered between the 95% limits of agreement, which suggests that results close to the observed values were obtained. The predicted values are more concentrated between these two lines compared to the other models.

Figure 9 displays the Bland-Altman plots for the prediction models based on the observed values for the Oslo station. Upon examining the figure, it is observed that the differences in the predicted values for the SVM-GA-M03 and SVM-PSO-M04 models are concentrated around zero. This indicates that these models yield more effective results compared to the others. The results here are consistent with the statistical findings.

To ensure the reliability of the results obtained in this study, ANOVA (Analysis of Variance) and Kruskal-Wallis statistical tests were applied to the forecasting models developed for the Stavanger, Kristiansand, and Oslo regions. The findings were examined at a 95% significance level in Table 4. For all stations, p-values were greater than 0.05, indicating that we fail to reject the null hypothesis of no statistically significant differences among the compared groups at 95% confidence. This suggests that the performance differences among the best models are modest and should be interpreted alongside the multi-metric evaluation and visual diagnostics. Also supporting the claim that while PSO-M04 had the highest metrics in general, its edge was not statistically significant.

**Table 4:** Statistical significance levels of the most successful models.

	F	Observed significance	H0
Stavanger			
Anova	0.2151	0.7972	Not Rejected
Kruskal-Wallis	-	0.7552	Not Rejected
Kristiansand			
Anova	0.1845	0.7925	Not Rejected
Kruskal-Wallis	-	0.7816	Not Rejected
Oslo			
Anova	0.1487	0.8125	Not Rejected
Kruskal-Wallis	-	0.7498	Not Rejected

## Discussion

The use of machine learning and optimization techniques in a hybridized manner is among the methods preferred by researchers across many disciplines in literature. Model performance metrics are typically improved using optimization techniques. In this study, consistent but moderate improvements in model performance values were also achieved through optimization techniques. The results obtained in this study, both in terms of the methods used and the optimization techniques applied, are consistent with those of several studies in literature. Some of these include: AlMohimeed et al.^116^ developed prediction models for the forward-looking estimation of cancer cells by utilizing image processing methods. The SVM-PSO model achieved one of the successful results. Just as this hybrid method was effective in the forward-looking prediction of cancer cells, it has also proven effective in the early diagnosis of hypertension problems^117^. In addition to their use in the health sector, hybrid methods are also utilized in hydrological studies. Oruc et al.^38^ investigated the performance of forward-looking drought prediction models in the Norwegian region by utilizing The Adaptive Neuro-Fuzzy Inference System (ANFIS) and SVM. They improved the model performances by hybridizing the machine learning algorithms with GWO, ABC, PSO, and GA. In their results, they emphasized that effective outcomes were obtained in analyses using SVM-PSO. In addition to these, they created 12 different model input structures using cross-correlation. In their conclusions, they stated that the data type and the number of delayed data points in the model input structure should be kept at an optimum level. Results consistent with these findings were also obtained in this study. To broaden the scope of the study, the model input structure in this work was created using the MRMR method instead of cross-correlation. Another study that obtained effective results with SVM-PSO is that of Samantaray et al.^118^. In their study, they chose Back Propagation Neural Network (BPNN) and SVM, along with PSO from the optimization techniques. Samantaray et al., who aimed to model the forward-looking prediction of floods in the Barak valley by creating 5 different input structures, obtained the most effective results from analyses performed with SVM-PSO. Unlike this study, they did not use any delayed data in their model input structures. Furthermore, they enriched their model input structure with meteorological data. In the results of their work, they also mentioned that effective results were achieved through this enrichment.

In many studies in the literature where hybrid machine learning and optimization techniques are preferred, usually only two or three optimization techniques are used. In this study, however, SVM, which is considered a well-established machine learning algorithm, was chosen and hybridized with four different optimization techniques (ABC, GWO, GA, and PSO). While these optimizers have been widely applied across different domains, side-by-side comparisons within a unified high-latitude PV forecasting setup remain relatively limited. Although the combined use of these methods strengthens the comparative aspect of our study, including additional machine learning models (e.g., a neural network or ensemble method) could further broaden the scope of comparison. However, such analyses involving optimization techniques are highly time-consuming and computationally expensive. This would move beyond the scope of this study and potentially become a topic for a separate follow-up work. Researchers interested in hybrid models may consider comparing a small set of learners and optimizers under consistent validation protocols and may also investigate sensitivity to learning rates and hyperparameter ranges.

The use of hybrid methodologies has become widespread across many disciplines today. Recently, deep learning techniques such as CNN, and CNN-LSTM have gained increasing visibility in research. These methods are considered highly innovative due to their nature as modifications of ANN architecture^119^. However, in this study, a key motivation was to benchmark the extent to which an established method (SVM) can benefit from input-structure design and metaheuristic tuning in a high-latitude PV forecasting context before moving to more complex deep-learning approaches. Deep-learning methods remain a promising direction for future work.

The models utilized in this study are data-driven models. Although data-driven models have advanced beyond physical-based models, the performance comparison between the two modeling approaches is frequently debated among researchers. This debate arises from the fact that physical-based models incorporate multiple parameters that accurately portray real-life phenomena. In contrast data-driven models can be very powerful but they also rely on heavily the quality and quantity of available data and sometimes may not capture certain physical constraints. Therefore, for critical applications, both approaches should be used to cross-validate results and ensure robustness for the area being examined.

Limitations of this study include: (i) daily aggregation of PV output, which smooths intra-day dynamics and ramp events; (ii) the use of a fixed 7-hour daytime window, which may not fully represent seasonal daylight variability at high latitudes; (iii) reliance on PVGIS-ERA5 derived PV output rather than site-measured power, which can introduce modeling biases; (iv) a restricted predictor set (primarily temperature and lagged PV) without additional meteorological drivers (e.g., irradiance, cloud cover, wind); (v) a limited number of sites and years (2020–2023); and (vi) evaluation based on a single chronological split without rolling-origin cross-validation or probabilistic forecasts.

These limitations restrict generalization beyond the studied sites and period. Future work should (i) use measured PV data when available, (ii) expand predictors, (iii) benchmark against additional ML/DL baselines (e.g., RF, gradient boosting, LSTM/GRU, CNN-LSTM), (iv) apply rolling-origin evaluation, and (v) report prediction intervals to quantify forecast uncertainty. Addressing these limitations would allow to generalize the findings.

## Conclusion

This study developed SVM-based hybrid models tuned with ABC, GWO, GA, and PSO to forecast daily PV power for three southern Norway locations (Kristiansand, Stavanger, and Oslo) using PVGIS-ERA5 derived PV output and temperature data covering 2020–2023. A chronological 70/30 train–test split was used to evaluate out-of-sample performance.

- Across all sites and input configurations, metaheuristic tuning improved test performance relative to the untuned SVM baseline. PSO produced the best mean test metrics, although differences among the top-performing optimizers were generally small.
- Input configurations that included both lagged PV power and lagged temperature (M03–M04) typically outperformed PV-only structures (M01–M02), confirming the value of incorporating meteorological inputs in PV forecasting.
- Model skill varied by location, with the highest scores obtained for Kristiansand in this dataset. These results should be interpreted as case-study findings for southern Norway rather than universal performance rankings.
- Visual error analysis (e.g., Bland–Altman plots) plots were particularly helpful for diagnosing bias and agreement between observations and predictions; however, when model metrics are very close, visual methods alone may be insufficient to clearly distinguish models.
- However, this study’s scope is limited to daily data from three sites and a specific train-test split; thus, the results should be interpreted as a case study rather than generalized truths.
- The use of simulated PVGIS data, a restricted set of input features, and absence of cross-validation can be list as key limitations in this study and will be addressed in future work.

Overall, the proposed SVM–metaheuristic framework provides a benchmark for high-latitude PV power forecasting. It demonstrates that even established machine learning models can benefit significantly from intelligent input selection and parameter tuning. Future studies can build on this by incorporating additional data sources (e.g., irradiation and cloud cover measurements) and exploring advanced models to further improve forecast reliability.

The hyper-parameters used for each technique are shown in Table 5.

**Table 5:** Hyperparameters used for each technique.

Algorithm	Hyper-parameter	Values
SVM	C	4
	γ	5
	Kernel Type	Linear
PSO	Swarm Size (N)	40
	Inertia Weight (w)	0.75
	Cognitive Coefficient (c1​)	2
	Social Coefficient (c2​)	2
GA	Population Size	120
	Crossover Probability (Pc​)	0.6
	Mutation Probability (Pm​)	0.1
GWO	Population Size (N)	40
	a Vector (Control Parameter)	1
	Maximum Iterations (T)	150
ABC	Colony Size	50
	Maximum Iterations (T)	50

## Data Availability

The original contributions presented in the study are included in the article. The raw data supporting the conclusions of this article will be made available by the corresponding author upon reasonable request.
